# Medium Optimization and Fermentation Kinetics for κ-Carrageenase Production by *Thalassospira* sp. Fjfst-332

**DOI:** 10.3390/molecules21111479

**Published:** 2016-11-05

**Authors:** Juanjuan Guo, Longtao Zhang, Xu Lu, Shaoxiao Zeng, Yi Zhang, Hui Xu, Baodong Zheng

**Affiliations:** 1 College of Food Science, Fujian Agriculture and Forestry University, Fuzhou 350000, Fujian, China; gjjfst15@163.com (J.G.); zlongtao@hotmail.com (L.Z.); luxuluxu88@163.com (X.L.); zsx1111@163.com (S.Z.); zyifst@163.com (Y.Z.); xhuifst@163.com (H.X.); 2 Fujian Provincial Technical Research Center of Marine Food and Biological Products Processing, Fujian Agriculture and Forestry University, Fuzhou 350000, Fujian, China

**Keywords:** κ-carrageenase, *Thalassospira* sp., response surface methodology, kinetics model

## Abstract

Effective degradation of κ-carrageenan by isolated *Thalassospira* sp. fjfst-332 is reported for the first time in this paper. It was identified by 16S rDNA sequencing and morphological observation using Transmission Electron Microscopy (TEM). Based on a Plackett–Burman design for significant variables, Box–Behnken experimental design and response surface methodology were used to optimize the culture conditions. Through statistical optimization, the optimum medium components were determined as follows: 2.0 g/L κ-carrageenan, 1.0 g/L yeast extract, 1.0 g/L FOS, 20.0 g/L NaCl, 2.0 g/L NaNO_3_, 0.5 g/L MgSO_4_·7H_2_O, 0.1 g/L K_2_HPO_4_, and 0.1 g/L CaCl_2_. The highest activity exhibited by *Thalassospira* sp. fjfst-332 was 267 U/mL, which makes it the most vigorous wild bacterium for κ-carrageenan production. In order to guide scaled-up production, two empirical models—the logistic equation and Luedeking–Piretequation—were proposed to predict the strain growth and enzyme production, respectively. Furthermore, we report the fermentation kinetics and every empirical equation of the coefficients (α, β, *X*_0_, *X_m_* and μ_m_) for the two models, which could be used to design and optimize industrial processes.

## 1. Introduction

Carrageenan, as a series of hydrophilic sulfated galactan multipolymers, is extracted from the red seaweed (*Rhodophyceaea*). Carrageenan consists of d-galactose residues linked by alternating linear chains of β-1,3-d-galactose (G) and α-1,4-linked-d-galactose (DA) [1]. Based on the number and position of sulfate ester units (S) and the presence of 3,6-anhydro galactose-bridges, carrageenan can be classified into κ-carrageenan (DA-G4S), ι-carrageenan (DA2S-G4S) and λ-carrageenan (D2S6S-G2S) [2]. κ-Carrageenan is composed of consecutive disaccharide units of β-d-galactose-4-sulfate and 3,6-anhydro-α-d-galactose, and it has been broadly applied in food, medicines, and biomaterials.

Carrageenan oligosaccharides are typically obtained through chemical hydrolysis [3], radiation [4] or carrageenase degradation, and they were reported to show potential bioactivities including anticoagulant, antithrombotic, antitumor and immunoregulation [3]. Chemical hydrolysis is the most common method to prepare carrageenan oligosaccharides. However, the method may cause damage to sulfate ester units and produce many complicated by-products, which limits it to industrial application. The enzymolysis approach has become a hot topic in research due to its high efficiency, specificity, and mild reaction conditions. According to this method, the main hydrophilic products are disaccharide, tetrasccharide, hexose, and octasaccharide [5]. Many carrageenases have been reported to be isolated from marine bacteria such as *Alteromonas* [6], *Cytophaga* [7], *Vibrio* [8], *Pseudomonas carrageenanovora* [9], and *Cellulophaga* [10]. However, the low activity and yield, along with the instability of the carrageenases, limit the utilization of this process for fermenting carrageenan oligosaccharides. Therefore, it is imperative to search for a bacterial strain that could produce carrageenase steadily and efficiently. 

Recently, physical induction or gene modification has been taken to improve the biological fermentation of carrageenase. Kobayashi cloned a gene from original bacteria *Pseudoalteromonas tetraodonis* JAM-K142 for an enzyme named as Cgk-K142, but the productivity of Cgk-K142 was rather low and the enzyme was very unstable during purification [11]. Another recombinant bacterium, GgkZ, yielded complex products which were difficult further purify [1]. Another method to enhance the bacterial enzyme yield is by adding an enzymatic inducer, which is also a low-cost and valid approach to improve the microbial fermentation. For example, lactose was found to increase the ability of recombinant bacteria BL21-HTa-cgkZ to degrade κ-carrageenan, with enzyme activity increased up to 10.76 U/mL after the optimization [12]. Agarase could be induced by agarose, neoagarobiose, neoagarotetraose and neoagarohexaose, but was inhibited by d-glucose and d-galactose [13]. Meanwhile, maltose was the most useful inducer for *Aspergillus niger* to yield glucoamylase [14], so is worthwhile to screen for proper inducers for marine bacteria fermenting carrageenase.

In this study, a novel strain that could effectively hydrolyze κ-carrageenan has been isolated from dry *Chondrus crispus* for the first time. We attempted to increase the κ-carrageenase activity by statistical optimization and determining the fermentation kinetics model. In addition, two empirical models were proposed for designing and optimizing the industrial process in varied environments, which were simulated under different fermentation conditions.

## 2. Results and Discussion

### 2.1. Isolation and Identification of κ-Carrageenase-Producing Bacterium

**Figure 1:**
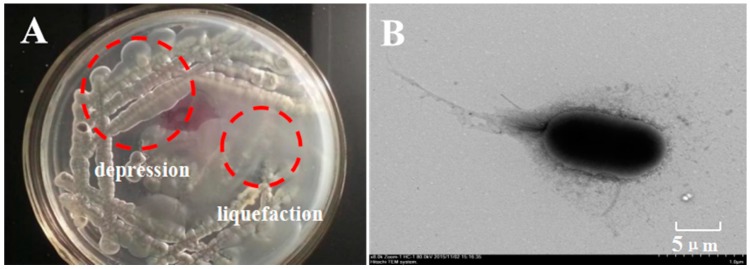
(**A**) The appearance of liquefaction and depression on plate medium and (**B**) the TEM image of *Thalassospira* sp. Fjfst-332.

**Figure 2:**
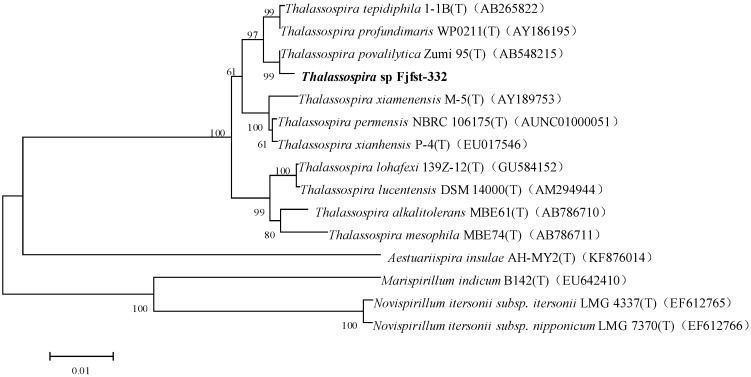
Phylogenetic tree of *Thalassospira* sp. Fjfst-332 based on 16S rDNA sequences and Neighbor Joining analysis.

κ-Carrageenan, selected as the only carbon source, was used to isolate a potential bacterium which could degrade κ-carrageenan effectively. Thereinto, the strain fjfst-332 exhibited the maximum liquefaction and depression activity on the plate medium (Figure 1). The physiological and biochemical characteristics (Table 1) were investigated according to Bergey’s Manual and were analyzed by TEM (Figure 1). The PCR amplification of the 16S rDNA gene yielded a 1487-bp sequence of the strain fjfst-332. The 16S rDNA sequence of fjfst-332 showed 98.7% similarity to *Thalassospira* sp. (Figure 2) based on the public databases (GenBank Accession Number EU440790). Hence, it was concluded that the strain was *Thalassospira* sp. and numbered as Fjfst-332, which is a novel κ-carrageenan-degrading species.

### 2.2. The Effect of Carbon Sources on the Enzyme Activity

**Figure 3:**
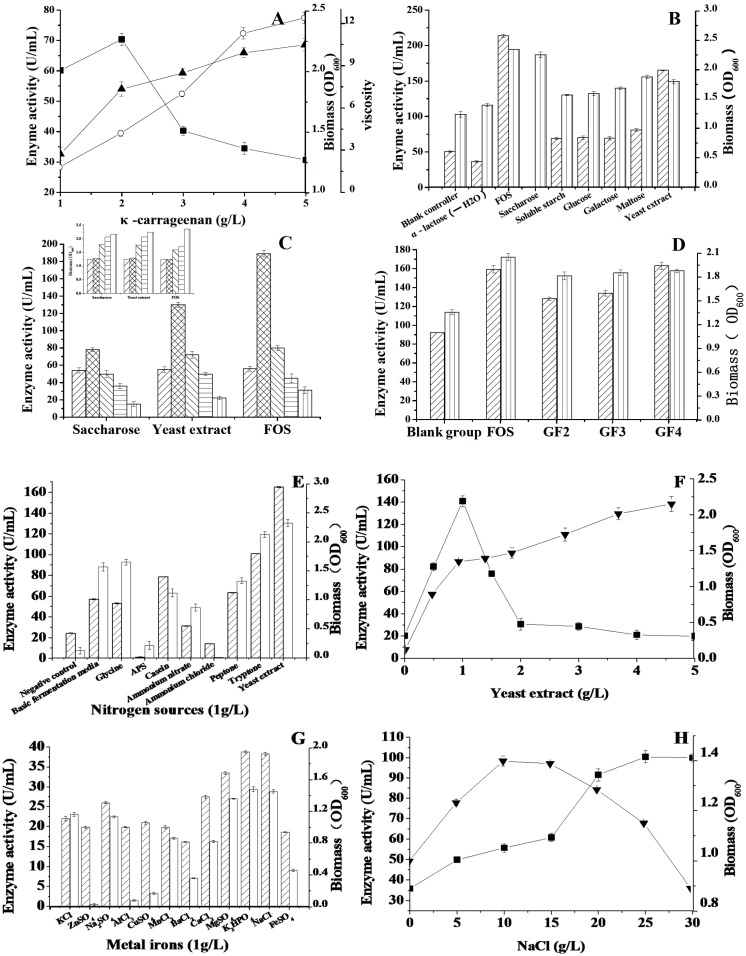
(**A**) Effects of κ-carrageenan on κ-carrageenase and biomass. ■ Enzyme activity, ▼ Biomass, ○ Viscosity; (**B**) Effects of other carbon sources on κ-carrageenase and biomass, including α-lactose (-H_2_O), FOS, saccharose, soluble starch, glucose, galactose, maltose and yeast extract with the concentration of 1 g/L, respectively. 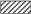 Enzyme activity, 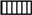 Biomass; (**C**) Effects of saccharose, yeast extract and FOS on κ-carrageenase and biomass (the small figure on the left). 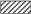 0 g/L, 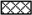 1 g/L, 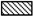 2 g/L, 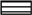 3 g/L, 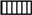 4 g/L; (**D**) Effects of inducers on κ-carrageenase and biomass, including FOS, GF2, GF3, GF4 with the concentration of 1 g/L. 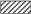 Enzyme activity, 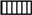 Biomass; (**E**) Effects of nitrogen sources on κ-carrageenase and biomass, the test nitrogen sources with final concentration of 1 g/L 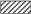 Enzyme activity, 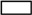 Biomass; (**F**) Effects of yeast extract on κ-carrageenase and biomass, ■ Enzyme activity, ▼ Biomass; (**G**) Effects of metal irons on κ-carrageenase and biomass, all of the metal irons with the concentration of 0.2 g/L, 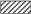 Enzyme activity; 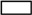 Biomass; (**H**) Effects of NaCl on κ-carrageenase and biomass, ■ Enzyme activity, ▼ Biomass.

The results (Figure 3A) showed that the concentration of κ-carrageenan could impact the effectiveness of κ-carrageenase production by strain fjfst-332 [15]. In Figure 3A, the maximum activity was observed at 2.0 g/L κ-carrageenan. There was a sharp decrease in κ-carrageenase activity with further increase of κ-carrageenan concentration, probably because a high concentration of κ-carrageenan could cause excessive viscosity (Figure 3A) and restrict normal bacterial growth. Other carbon sources, shown in Figure 3B, also influenced the growth status and enzyme activity of strain fjfst-332. On a base of 2.0 g/L κ-carrageenan, monosaccharides, disaccharides, and soluble starch were able to multiply strain growth, but the enzyme activities were not affected by these carbon sources. Interestingly, FOS, saccharose, and yeast extract could enhance the biomass and enzyme activity of strain fjfst-332, respectively (Figure 3B). Yeast extract has generally been used to incubate bacteria, and FOS had been found to multiply on bifidobacterium [16,17]. Micromolecule substances, such as sophorose, cellobiose, and lactose were easily observed during the fermentation, especially with the enzyme production [18]. As shown in Figure 3C, 1 g/L FOS could induce strain fjfst-332 to yield κ-carrageenase effectively, in amounts almost three fold higher than the blank group. What’s more, we also researched the influence of specific components of FOS on κ-carrageenase activity (Figure 3D). GF4, the typical composition of FOS, was the optimal component for the enzyme activity of the isolated bacterium. In other reports, Zhou has published that glucose was the supplementary carbon resource for strain WZUC10 to degrade κ-carrageenan, but it did not explain the mechanism for carrageenase expression in microorganisms [2]. In our study, how the FOS or GF4 regulates gene expression and producing κ-carrageenase in strain fjfst-332 is not clear, yet. The mechanism needs further research.

### 2.3. The Effects of Nitrogen Sources, Metal Ions and NaCl on the Enzyme Activity

Both organic and inorganic nitrogen influenced the strain growth, and the nitrogen sources were studied based on 2.0 g/L κ-carrageenan. As shown in Figure 3E, yeast extract was the most suitable nitrogen source for κ-carrageenase production. The cell biomass was enhanced by increasing the yeast extract concentration from 0 g/L to 5g/L (Figure 3F). The maximum activity was observed at 1 g/L yeast extract. However, when the concentration of yeast extract exceeded 1 g/L, the activity of κ-carrageenase declined. Hence, the growth of the bacteria was induced by yeast extract, but the activity of κ-carrageenase did not increase with the same trend. The results were similar to findings on *Vibrio* sp. strain JT01017 [15], where polypeptone and yeast extract had the same effect on the growth of the strain and the production of agarase. It also could be observed that when the ratio of κ-carrageenan and yeast extract was lower than 2:1, the activity of κ-carrageenase obviously declined. The reason may be that the κ-carrageenase was an induced enzyme for strain *Thalassospira* sp. Fjfst-332, which only could be induced by the κ-carrageenan. When the concentration of yeast extract was high, the κ-carrageenan-degrading function would be inhibited because of substrate competition.

The possibility of an artificial sea water impact on κ-carrageenase production and cell growth was considered [19]. NaCl, NaNO_3_, MgSO_4_, 7H_2_O, K_2_HPO_4_, and CaC1_2_ were found to have significant effects on catalyzing κ-carrageenan (Figure 3G). The results were consistent with a previous report by Mou [20], showing the influence of these ions on κ-carrageenase production. In addition, the concentration of NaCl also made a difference in the bacterial growth and the κ-carrageenan hydrolysis function. The optimal concentration of NaCl was found to be 25 g/L (Figure 3H). The effect of NaCl on strain fjfst-332 was similar to its effect on *Pseudoalteromonas*-like bacterium WZUC10 [2] and *Pseudoalteromonas* sp. QY203 [21], where 25 g/L was the optimal concentration. Nevertheless, there are also opposite conclusions showing that κ-carrageenase did not require NaCl for activity. Wang reported that a new recombinant κ-carrageenase CgkS exhibited a high specific activity to κ-carrageenan in the absence of NaCl [1]. 

### 2.4. Screening of Significant Variables by Plackett–Burman (PB) Design

**Figure 4:**
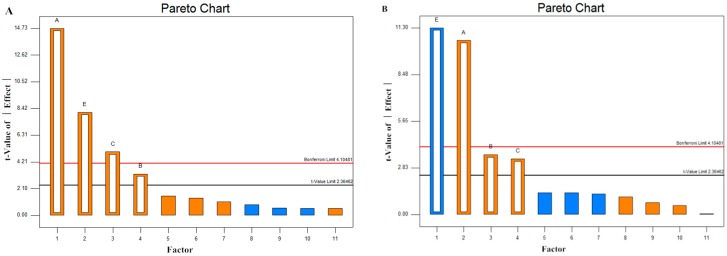
Pareto Chart of Plackett-Burman design for enzyme activity (**A**) and Biomass (**B**). A: κ-carrageenan, B: FOS, C: tryptone, E: NaCl.

A two-level PB design with 11 runs was arranged as shown in Table 2, and the results are shown in Table 3. Figure 4 illustrates the Pareto Chart (*p* < 0.05) of significant effects. The *t*-values of κ-carrageenan, NaCl, yeast extract, and FOS were better than the *t*-value limit (*t* > 2.36462), which confirmed that the predicted value was significant and acceptable. According to the PB and the Pareto Chart regression results, the lack of significant dummy factors for any of the responses indicated that there were neither unknown variables nor systematic errors in the PB design. With respect to enzyme activity, κ-carrageenan, NaCl, yeast extract, and FOS had significant effects (Figure 4A). With respect to biomass, NaCl demonstrated significant negative effects (Figure 4B), which could mean that the strain was sensitive to salt and low NaCl was ideal. In addition, κ-carrageenan, yeast extract, and FOS were significant factors with positive effects on biomass (Figure 4B). 

In sum, κ-carrageenan, NaCl, yeast extract, and FOS were the significant variables for enzyme activity (EA) and biomass. The optimal concentrations were chosen for RSM design as follows: κ-carrageenan (1, 2, 3 g/L), NaCl (15, 20, 25 g/L), yeast extract (1, 2, 3 g/L), and FOS (1, 2, 3 g/L).

### 2.5. Model Statistical Analysis

The RSM modeling results for EA and BM are presented in Table 4. The model’s *R*^2^ values were 0.9689 (EA) and 0.9700 (BM), respectively, with a 95% confidence level. The experimental data fit the second order polynomial model (Equations (1) and (2)) well and described the relationship among κ-carrageenan, NaCl, yeast extract, and FOS. Furthermore, the lack of fit was non-significant (EA 0.0888, BM 0.0598), indicating that the model was acceptable (Table 5).

EA = 266.19 − 11.23*X*_1_ + 7.06*X*_2_ − 11.57*X*_3_ − 9.06*X*_4_ + 22.73*X*_1_*X*_2_ − 27.38*X*_1_*X*_3_ − 41.04*X*_1_*X*_4_ + 14.87*X*_2_*X*_3_ + 1.91*X*_2_*X*_4_ − 44.73*X*_3_*X*_4_ − 22.03*X*_1_^2^ − 23.43*X*_2_^2^ − 41.11*X*_3_^2^ − 49.78*X*_4_^2^ (1)

BM = 0.52 + 0.010*X*_1_ − 0.018*X*_2_ + 0.0007833*X*_3_ + 0.00075*X*_4_ − 0.0009750*X*_1_*X*_2_ + 0.00075*X*_1_*X*_3_ − 0.013*X*_1_*X*_4_ + 0.00005*X*_2_*X*_3_ + 0.012*X*_2_*X*_4_ + 0.00005*X*_3_*X*_4_ − 0.032*X*_1_^2^ − 0.082*X*_2_^2^ − 0.025*X*_3_^2^ − 0.013*X*_4_^2^ (2)

### 2.6. Effect of Independent Variables on Enzyme Activity (EA) and Biomass (BM)

The positive linear effects of X_1_ (κ-carrageenan) and X_3_ (yeast extract) on EA were found to be highly significant (*p* < 0.01), and the effects of X_2_ (NaCl) and X_4_ (FOS) were significant (*p* < 0.05), which agrees with the conclusion of the PB analysis. Furthermore, all the quadratic effects of X_1_^2^, X_2_^2^, X_3_^2^, and X_4_^2^ were found to be significant (*p* < 0.05) and negative in value, which accounts for the equation of the parabola function decreasing and the maximum point. The results of the analysis of variance (ANOVA) indicated that the reciprocities of X_1_X_2_, X_1_X_3_, X_1_X_4_, and X_3_X_4_ were highly significant (*p* < 0.01), and the reciprocity of X_2_X_3_ was significant (*p* < 0.05) for κ-carrageenase activity (Table 5). The strain biomass was most affected by κ-carrageenan and NaCl (*p* < 0.001), followed by yeast extract and FOS (*p* < 0.05). All of the quadratics were significant items (*p* < 0.01). The reciprocities of X_1_X_2_, X_1_X_3_, X_1_X_4_, and X_2_X_4_ were highly significant (*p* < 0.01), yet those of X_2_X_3_ and X_3_X_4_ were non-significant (*p* > 0.05).

**Figure 5:**
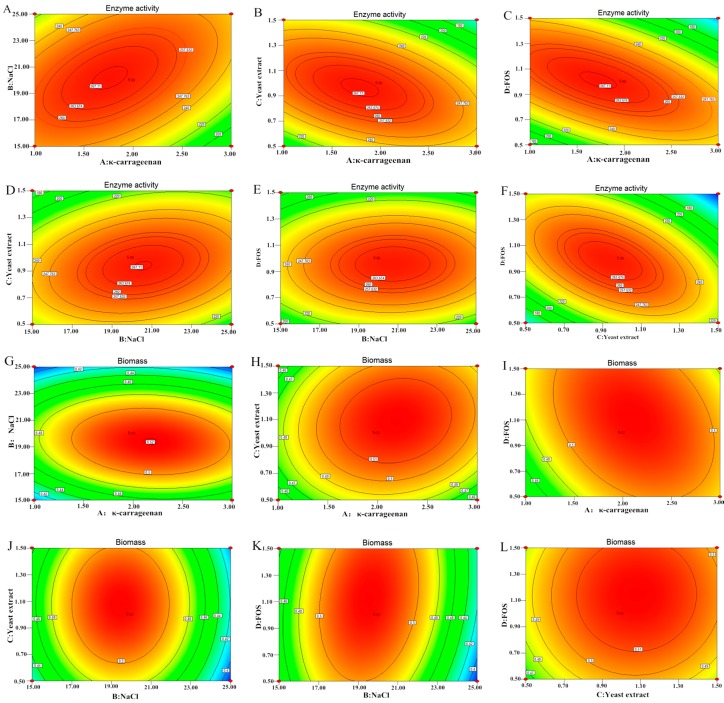
(**A**–**F**) was the interaction effect of variables on enzyme activity (EA); (**G**–**L**) was the interaction effect of variables on biomass (BM).

Enzyme activity increased with the four factors, with its peak activity approaching the midpoint of the response plot. The interaction of κ-carrageenan and NaCl was highly sensitive to EA, indicating that EA increased when they increased. However, high concentrations of κ-carrageenan and NaCl restrained EA (Figure 5A). Similarly, EA increased as yeast extract and FOS increased, but high concentrations led to low EA (Figure 5B,C,F). κ-carrageenase activity was negatively influenced by NaCl and FOS (Figure 5E). The biomass followed similar trends as EA (Figure 5G–L). A high concentration of NaCl inhibited BM but made enhanced EA, so the optimal amount was determined to be 20 g/L [2].

The RSM results showed that the ideal EA was 267.851 U/mL with the medium composition: 1.76 g/L κ-carrageenan, 20.04 g/L NaCl, 0.96 g/L yeast extract, and 1.02 g/L FOS. The maximum predicted value of BM was 0.521281 (OD_600_) at 2.17 g/L κ-carrageenan, 19.47 g/L NaCl, 1.09 g/L yeast extract, and 1.08 g/L FOS. Combined with the actual operation and Plackett–Burman design, the test was executed five times using the simplified medium: 2 g/L κ-carrageenan, 20 g/L NaCl, 1 g/L yeast extract and 1 g/L FOS, and the average EA was 265 ± 1.56 U/mL. Compared with bacteria investigated in previous reports—*Pseudoalteromonas* sp. WZUC10, 50 U/mL [2] and ALAB-001, 170 U/mL [8], strain fjfst-332 showed the highest crude enzyme activity (EA).

### 2.7. Verification Tests

**Figure 6:**
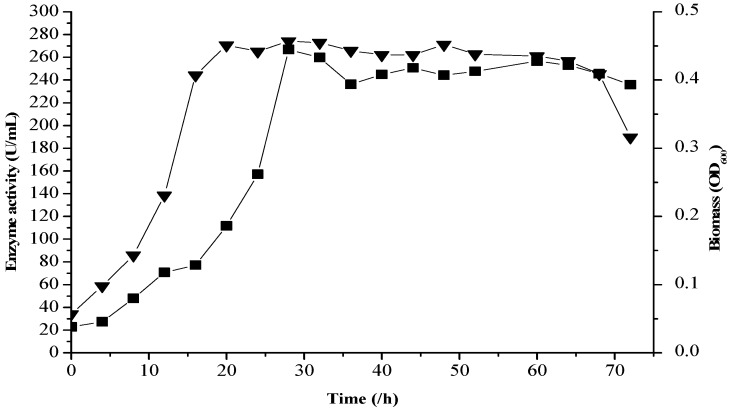
The cell growth (▼) and enzyme activity (■) curve of strain *Thalassospira* sp. fjfst-332.

To verify the reliability of the RSM models, the strain fjfst-332 enzyme production and cell growth curve were investigated under the optimized medium (Figure 6). Strain fjfst-332 reached the logarithmic phase at 20 h and increased to the stationary phase at 24 h. Consideration the fermentation forms, the fermentation model of strain fjfst-332 was part of growth-related type II (α ≠ 0, β ≠ 0). In detail, the fastigia of bacterial growth (20 h) and metabolite accumulation (28 h) were not synchronous, and the fastigium of biomass growth was reached faster than that of κ-carrageenase. After the stationary phase (32 h), the growth of metabolite (oligosaccharide) was smooth and steady, eventually accompanied by the catabolite stacking and κ-carrageenan consumption. The growth of κ-carrageenan oligosaccharide increased in stability in 58 h. After 62 h both the enzyme production and the cell biomass went into the decline phase. It might be the effect of substrate consumption. At the fastigium, the maximum EA and BM yields were 266.84 U/mL and 0.519 (OD_600_), respectively, which matched the predicted values with relative error less than 2%. Thus, the RSM regression models could predict the EA and BM yields for the medium composition of κ-carrageenan, NaCl, yeast extract, and FOS.

### 2.8. Fermentation Kinetics

**Figure 7:**
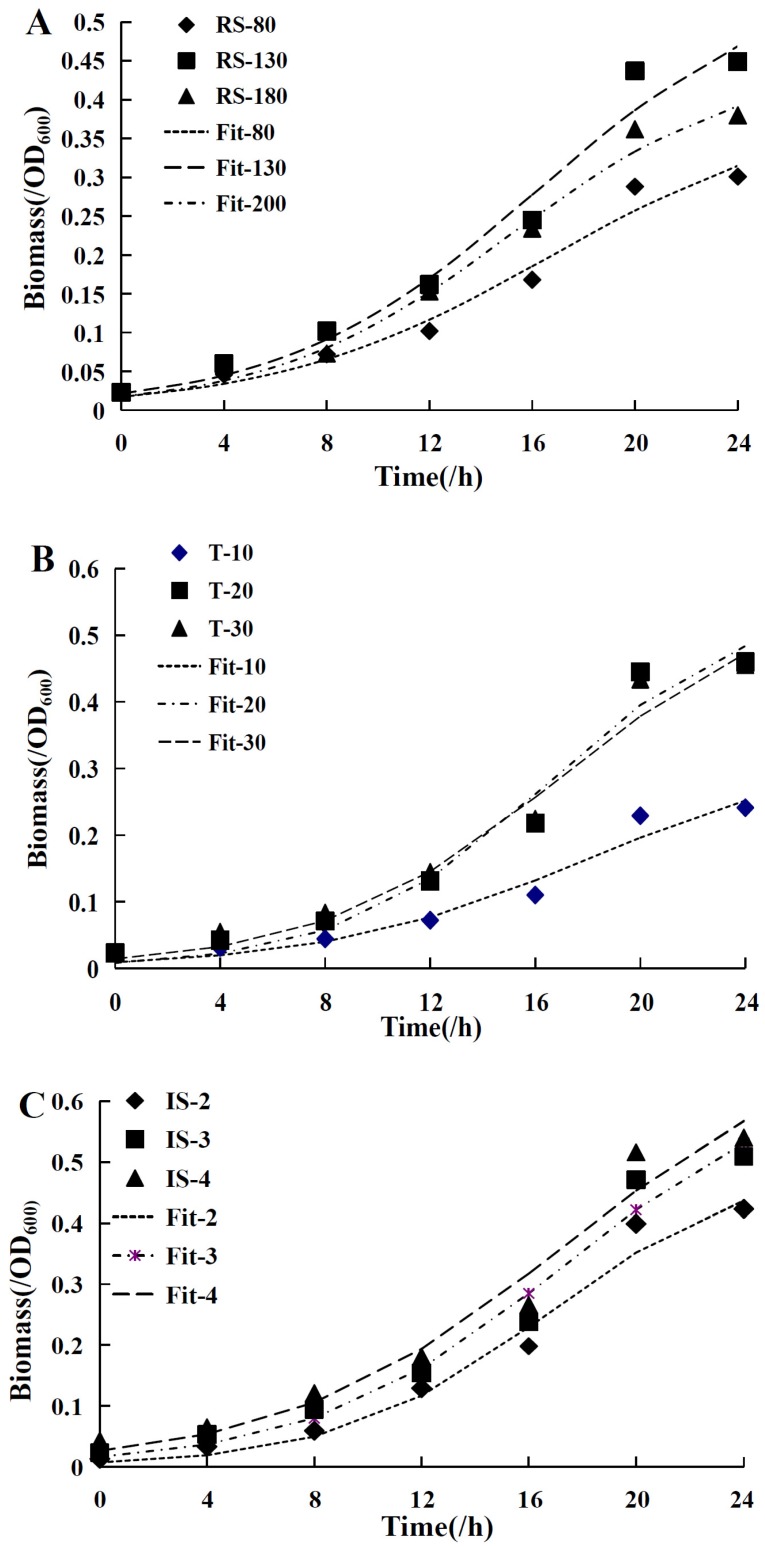
(**A**) is the model fitting of revolving speed relative to biomass, altering variables 80r/min, 130 r/min and 180 r/min, quantifying temperature 30 °C, and inoculum size 3 mL; (**B**) was the model fitting of temperature relative to biomass, altering variables 10 °C, 20 °C and 30 °C, quantifying revolving speed 130 r/min, and inoculum size 3 mL; (**C**) was the model fitting of inoculum size relative to biomass, altering variables 2 mL, 3 mL and 4 mL, quantifying revolving speed 130 r/min and temperature 30 °C.

In order to investigate the cell growth curve and product formation curve of strain fjfst-332, the logistic equation and Luedeking–Piret equation were utilized to predict the experimental data, which are shown in Figure 7. The traditional fitting method appeared to be deficient because of only for each individual case, while the empirical model could be applied in various environments.

### 2.9. Logistic Cell Growth Model

The non-linear regression analysis was used to estimate the logistic model, and the data are shown in Table 6. Under the different conditions (stirring speed, temperature, and bacterial load), the parameters (*X*_0_, *X_m_*, μ*_m_*) varied. The results showed that all of the *R*^2^ > 0.96, indicating that the experimental data had a high degree of fit with the logistic equation. Furthermore, different conditions (RS, T, and IS) led to different parameters (*X*_0_, *X_m_*, μ*_m_*) in accordance with the logistic equation. SPSS software was used to fit the nonlinear regression connection of parameters with RS, T, and IS, which conformed to the polynomial equation. The empirical models are demonstrated in Equations (4)–(6). Establishing a system model to investigate cell growth in all kinds of fermentation environments would be well utilized in directing scale production (Figure 7). Goudar created a mathematical model according to logistic equations, which were helpful in the development of the early- and late-stage fed-batch process [22]:

(3) $$X=\frac{X_{0}X_{m}e^{\mu_{m}t}}{X_{m}-X_{0}+X_{0}e^{\mu_{m}t}}$$

*X*_0_ = 0.00004 − 0.000036I − 0.00000628T + 0.000053T^2^ − 0.00000018R^2^ (4)

*X_m_* = −0001 − 0.002I + 0.002T + 0.006R − 0.002I × T + 0.005I^2^ − 0.001T^2^ − 0.000052R^2^ (5)

*U_m_* = −0.0000705 − 0.0000803I + 0.003T + 0.005R + 0.004I × T − 0.002I × R + 0.000044T × R + 0.012I^2^ − 0.001T^2^ − 0.0000036R^2^ (6)

### 2.10. The Luedeking–Piret Model

**Figure 8:**
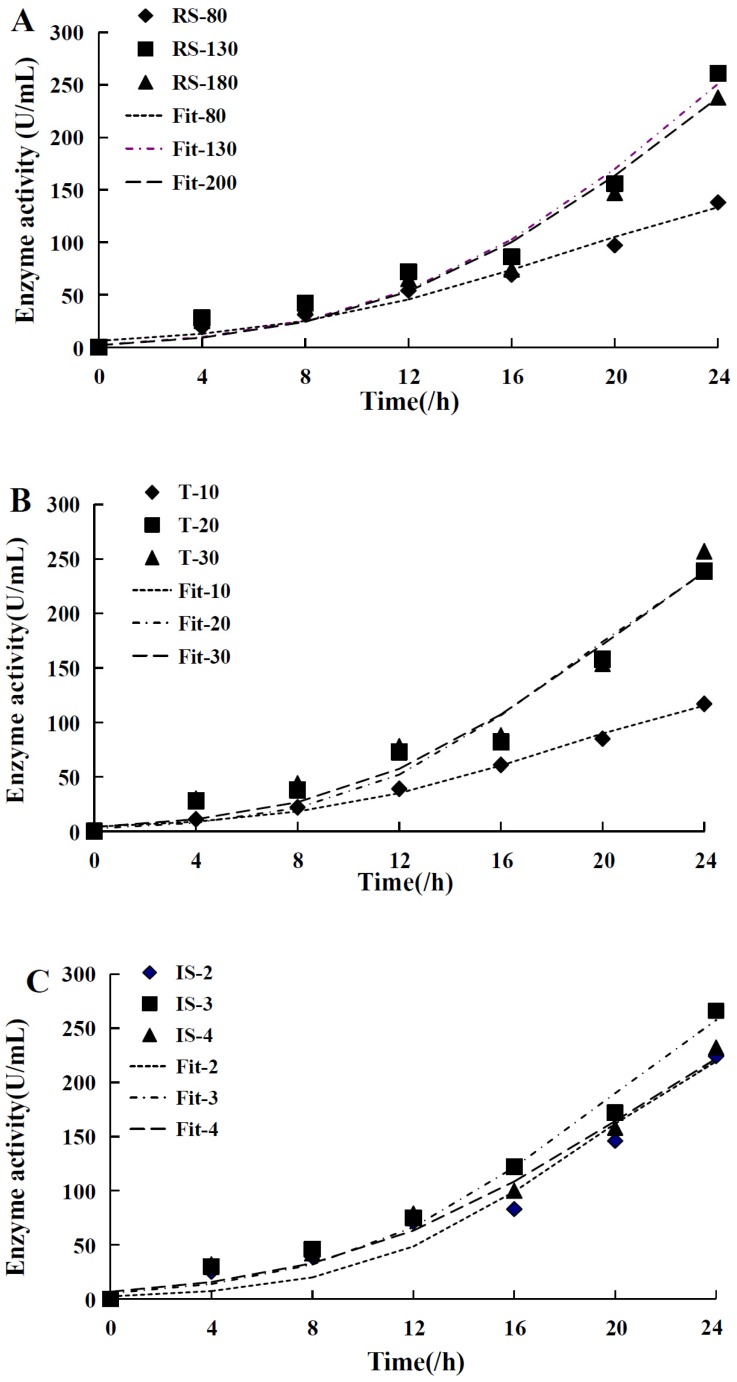
(**A**) was the model fitting of revolving speed relative to enzyme activity, altering variables 80 r/min, 130 r/min and 180 r/min, quantifying temperature 30 °C, and inoculum size 3 mL; (**B**) was the model fitting of temperature relative to enzyme activity, altering variables 10 °C, 20 °C and 30 °C, quantifying revolving speed 130 r/min, and inoculum size 3 mL; (**C**) was the model fitting of inoculum size relative to enzyme activity, altering variables 2 mL, 3 mL and 4 mL, quantifying revolving speed 130 r/min and temperature 30 °C.

The Luedeking–Piret equation is a nonlinear regression equation used to simulate the relationship between bacterial growth concentration and product formation [23]. The verification test was carried out at optimal medium (fermentation conditions 125 r/min, 25 °C, and 3 mL inoculum size), and preliminary analysis was performed on the batch pattern owing to the growth type (II). The results were shown in Table 6. Particularly, the coefficients of α ≠ 0, β = 0 were fitted under the condition of 10 °C, which showed that the cell growth concentration and product formation pattern fell under growth type I (α ≠ 0, β = 0) and *R*^2^ > 0.990. In other conditions, the fermentation patterns were all type II, and *R*^2^ > 0.960 (Figure 8). This pattern has been seen infrequently in other reports. The explanation for the results may be that κ-carrageenan was easily coagulated at low temperature, so that it influenced the cell growth normally. Finally, the empirical models regarding α, β with *X*_0_, *X_m_*, and μ_*m*_ were simulated using a polynomial equation in the same way (Equations (9) and (10)):

(7) $$\frac{dP}{dt}=\alpha \frac{dX}{dt}+\beta X$$

(8) $$P=\alpha \frac{X_{0}X_{m}e^{\mu_{m}t}}{X_{m}-X_{0}+X_{0}e^{\mu_{m}t}}+\beta \frac{X_{m}}{\mu_{m}}\ln\left(\frac{X_{m}-X_{0}+X_{0}e^{\mu_{m}t}}{X_{m}}\right)$$

α = −4.15 − 0.91I − 0.18T + 7.21R − 4.72I × T − 2.29I ×R − 0.12T × R + 66.84I^2^ + 0.47T^2^ + 0.004R^2^ (9)

β = 0.14 − 0.09I + 0.42T + 0.03R + 1.30I × T + 0.23I × R − 0.001T × R − 11.78I^2^ − 0.07T^2^ − 0.001R^2^ (10)

## 3. Materials and Methods

### 3.1. Chemicals and Materials

κ-Carrageenan (BR) and fructo-oligosaccharide (BR) were purchased from Solarbio Company (Beijing, China); Kestose (GF2), nystose (GF3) and GF4 were purchased from Sigma-Aldrich (St. Louis, MO, USA); other chemical reagents were all BR grade.

### 3.2. Bacterial Isolation

The dried fragments of red seaweed *Chondrus ocellatus* (Zhangzhou Lvqi Colloid Food Company of Fujian Province, Zhangzhou, China) that is the raw material for extracting carrageenan was chosen to isolate κ-carrageenan-hydrolyzing bacterial strains. The plate medium (15 g/L NaCl, 15 g/L κ-carrageenan, 2 g/L NaNO_3_, 0.5 g/L MgSO_4_·7H_2_O, 1 g/L K_2_HPO_4_, 0.l g/L CaC1_2_, pH = 7.5, 28 °C) was screened for bacterial colonies that manifested plate depression or liquefaction-forming activity. Pure cultures of the depression-forming bacterial isolates were obtained through repeated subculture at 28 °C for 24 h (the medium was supplemented by a nutritional ingredient with 5 g/L peptone).

### 3.3. Identification of Strain Fjfst-332

The cellular morphology and physiology of the strain were determined using a transmission electron microscope (TEM, JEM-1400 120kV, JEOL, Beijing, China) and Bergey’s Manual of Determinative Bacteriolog [24]. Genomic DNA was extracted using an Ezup Column Bacteria Genomic DNA Purification Kit (cat. NO. SK8255, Sangon Biotech, Shanghai, China). The 16S rDNA gene was amplified using the primer pair 7F (5′-CAGAGTTTGATCCTGGCT-3′) and 1540R (5′-AGGAGGTGATCCAGCCGCA-3′). The PCR products were sequenced and analyzed using a sequencing system (ABI 337 DNA, Model: 3730XL, Applied Biosystems, Foster City, CA, USA). Using the purified sequence as the query sequence, similar 16S rDNA sequence data were looked up in GenBank and downloaded from the NCBI online tool (http://rdp.cme.msu.edu/index.jsp; BLAST; Accession Number EU440790).

### 3.4. Selection of Significant Variables via Plackett–Burman (PB) Design

Based on the single-factor experiment results, the Plackett–Burman design [25] was used to investigate the influence of κ-carrageenan (A), FOS (B), tryptone (C), NaCl (E), K_2_HPO_4_ (F), MgSO_4_ (G), and CaCl_2_ (H) on the strain enzyme activity and biomass. Three dummy factors (D1, D2, D3) were introduced to investigate the systematic error.

### 3.5. Statistical Optimization

Many variables, such as the carbon source, nitrogen source, metal ions, etc., are known to affect enzyme activity and strain biomass. In the present study, the influence of κ-carrageenan and other auxiliary carbon sources was investigated. Organic and inorganic nitrogen sources were also investigated, and various valence metal ions were considered. Medium components with significant effects were screened based on PB experiments. All of the experiments for media components were carried out in triplicates.

A Box-Behnken experimental design and response surface methodology (RSM) were used to evaluate the relationship between the medium composition (κ-carrageenan, NaCl, yeast extract, FOS) and κ-carrageenase activity (EA). A three-level, four-factor Central Composite Design (CCD), consisting of 29 runs including 24 analytical points, was used. The four independent variables were arranged at three levels (−1, 0, +1) (Table 7). The test data were fitted to the generalized second order polynomial model equation (Equation (11)), which was used in the RSM analysis:

(11) $$Y=\beta_{0}+\sum_{i=1}^{k}\beta_{i}X_{i}+\sum_{i=1}^{k}\beta_{ii}{X_{ii}}^{2}+\sum_{i}^{k-1}\sum_{j}^{k}\beta_{ij}X_{i}X_{j}$$

where, *Y* was the response variable (EA); *X_i_* and *X_j_* were the independent variables. β_0_ was the constant coefficient, β*_i_* was the linear coefficient, β*_ii_* was the quadratic coefficient, and β*_ij_* was the cross product coefficient.

### 3.6. Evaluation of κ-Carrageenase Activity (EA)

κ-Carrageenase activity (EA) was determined by using the 3,5-dinitrosalicylic acid (DNS) method [26]. The incubation conditions were as follows: 5 mL κ-carrageenan solution (0.2% κ-carrageenan in deionized water) was degraded using 1 mL crude κ-carrageenase at 45 °C for 60 min, then mixed with 1 mL DNS; 1 mL resulting mixture was heated at 100 °C for 5 min. The absorbance of the mixture (10 times dilution) was read at 540 nm. One unit of κ-carrageenase activity (EA) is equivalent to an enzyme that produces 1 μg of d-galactose per minute under the given conditions. κ-Carrageenase activity was calculated using the formula:

(12) $$EA(U/mL)=\frac{(A\times 640.5+13.99)\times V\times n}{v\times T}$$

where A is the absorbance under OD_540_, 640.5 and 13.99 are the constants for calculating liberated galactose, V is the total reaction volume (mL), n is the dilution factor, v is the volume of enzyme (mL), and T is the length of digestion time (min).

### 3.7. Determination of Cell Biomass (BM)

Turbidimetry was used to determine the fjfst-332 strain biomass (BM). A 250 mL Erlenmeyer flask containing 30 mL optimized fermentation medium with 3 mL seed culture was incubated at 125 r/min and 30 °C for 72 h. Samples were taken every 4 h and the turbidity at OD_600_ was measured.

### 3.8. Detemination of Viscosity

A NDJ-7 Rotary Viscosimeter (Beijing ZKDH, Beijing, China) was used to determine the viscosity. The initial medium with 1 g/L~5 g/L κ-carrageenan were determined under the room temperature. The viscosity was calculated using the formula: η = kα, where η is the viscosity; k is the coefficient of the rotor; α is the reading number.

### 3.9. Mathematical Model of Fermentation Process

#### 3.9.1. Determination of Cell Growth Dynamics

In order to assess the growth pattern of strain fjfst-332, a logistic equation (Equation (13)) was used to predict the cell growth model at log phase and stable phase. The logistic equation describes the growth of a simple population in a confined space, where resources are limited [22]. Due to the bacteria having different growth curves in different fermentation environments [27], the dynamics equation was fitted at the conditions of revolving speed (RS 80 r/min, 130 r/min, 200 r/min), temperature (T 10 °C, 20 °C, 30 °C), and bacterial load (IS 2 mL, 3 mL, 4mL), based on the optimal medium:

(13) $$\frac{dX}{dt}=\mu_{m}X\left(1-\frac{X}{X_{m}}\right)$$

Equation (13) integrated into the following algebraic equation:

(3) $$X=\frac{X_{0}X_{m}e^{\mu_{m}t}}{X_{m}-X_{0}+X_{0}e^{\mu_{m}t}}$$

where *X*_0_ is the initial cell biomass, *X_m_* is the maximum cell biomass, μ*_m_* is the maximum specific growth rate, and *t* is the fermentation time.

#### 3.9.2. Analysis of Product Formation Kinetic

Gaden [28] generalized three fermentation forms: I. growth-related type; II. growth part-related type; III. non-growth-related type. Luedeking and Piret [29] proposed the Luedeking–Piret equation (Equation (7)) according Gaden’s conclusion, which considered the relationship of cell growth to product formation. When the cells, or some constituent of cells that is proportional to cell mass, are the product, the rate of product formation directly relates to the rate of growth. The relationship of product concentration with cell concentration can be rewritten into Equation (8):

(7) $$\frac{dP}{dt}=\alpha \frac{dX}{dt}+\beta X$$

where *P* is the product concentration, *X* is the concentration of cells, *t* is the fermentation time, and α and β are the coefficients. I: α ≠ 0, β = 0; II: α ≠ 0, β ≠ 0; III: α = 0, β ≠ 0.

(8) $$P=\alpha \frac{X_{0}X_{m}e^{\mu_{m}t}}{X_{m}-X_{0}+X_{0}e^{\mu_{m}t}}+\beta \frac{X_{m}}{\mu_{m}}\ln\left(\frac{X_{m}-X_{0}+X_{0}e^{\mu_{m}t}}{X_{m}}\right)$$

where *X*_0_ is the initial cell biomass, *X_m_* is the maximum cell biomass, μ*_m_* is the maximum specific growth rate, *t* is the fermentation time, and α and β are the coefficients.

### 3.10. Statistical Analysis

The statistical analysis systems DPS (V9.50, Ruifeng Information Company, Hangzhou, China) and Origin Pro (V8.5, OriginLab, Wellesley Hills, WA, US) were used to analyze the data. A one-factor analysis of variance (ANOVA) was performed for each parameter. Values given below are the means of repeatedly measured values. The PB and RSM analysis were performed using Design-Expert (V8.0.6, State-East Company, Minneapolis, MN, USA). The fitting of two kinetic models (cell growth model, logistic equation, and product formation model, Luedeking–Piret equation) to the experimental data of each sample was carried out using IBM SPSS Statistics (V21.0, IBM, Armonk, NY, USA). The *R*^2^ coefficient was used to evaluate the accuracy of the experimental data’s fit to the models.

## 4. Conclusions

The strain *Thalassospira* sp. fjfst-332 isolated from dry *Chondrus crispus* was shown for the first time to degrade κ-carrageenan. According to statistical analysis, the optimal fermentation components for strain fjfst-332 were determined to be 2 g/L κ-carrageenan, 20 g/L NaCl, 1 g/L yeast extract, 1 g/L FOS, 2 g/L NaNO_3_, 0.5 g/L MgSO_4_·7H_2_O, 1 g/L K_2_HPO_4_, and 0.l g/L CaC1_2_, resulting in the strain’s highest enzyme activity of 267 U/mL. Compared with previous reports, fjfst-332 was the most effective wild bacterium for obtaining κ-carrageenan oligosaccharides. We also identified an inducer (FOS) to make strain fjfst-332 more prolic and enable it to hydrolyze κ-carrageenan more effectively. What’s more, our research revealed three much more adaptive empirical models to describe the microorganism growth and product formation. All of this work will be useful as a foundation for the industrial production of κ-carrageenan oligosaccharide.

## Figures and Tables

**Table 1:** Physiological and biochemical characteristics.

Feature	Results	Items	Results
Morphological feature		Biochemical Characteristics	
mycelial morphology	rhabditiform	oxidase	−
gram stain	positive	hydrogen peroxide	−
spore	−	gelatin liquefaction	−
flagellum	+	amylolysis	+
Cultural Characteristics		carrageenan dispergation	+
colonial morphology	depression, adhesion		
bacterial colony	smooth		
borderline state	irregular		
pigment	faint yellow		
demand for oxygen	facultative anaerobic		

“+” represent positive; “−” represent negative.

**Table 2:** Arrangement and experimental results of the PB design.

NO.	A	B	C	D	E	F	G	H	D1	D2	D3	EA (U/mL)	BM, (OD_600_)
1	2.5	1.5	0.5	2	25	0.3	0.1	0.1	−1	1	−1	250.12 ± 1.27 ^a^	0.428 ± 0.005 ^c,d^
2	1	1.5	1.5	1	25	0.3	0.3	0.1	−1	−1	1	215.21 ± 1.17 ^f^	0.402 ± 0.006 ^e^
3	2.5	0.5	1.5	2	10	0.3	0.3	0.3	−1	−1	−1	234.12 ± 1.73 ^c^	0.473 ± 0.011 ^b^
4	1	1.5	0.5	2	25	0.1	0.3	0.3	1	−1	−1	181.25 ± 1.58 ^h^	0.39 ± 0.008 ^e,f^
5	1	0.5	1.5	1	25	0.3	0.1	0.3	1	1	−1	191.25 ± 2.93 ^g^	0.385 ± 0.009 ^f^
6	1	0.5	0.5	2	10	0.3	0.3	0.1	1	1	1	123.41 ± 1.38	0.418 ± 0.010 ^d^
7	2.5	0.5	0.5	1	25	0.1	0.3	0.3	−1	1	1	245.28 ± 0.72 ^b^	0.403 ± 0.008 ^e^
8	2.5	1.5	0.5	1	10	0.3	0.1	0.3	1	−1	1	221.32 ± 2.01 ^e^	0.471 ± 0.006 ^b^
9	2.5	1.5	1.5	1	10	0.1	0.3	0.1	1	1	−1	231.55 ± 1.48 ^d^	0.489 ± 0.012 ^a^
10	1	1.5	1.5	2	10	0.1	0.1	0.3	−1	1	1	164.25 ± 1.53 ^i^	0.431 ± 0.007 ^c^
11	2.5	0.5	1.5	2	25	0.1	0.1	0.1	1	−1	1	253.32 ± 1.77 ^a^	0.428 ± 0.010 ^c,d^
12	1	0.5	0.5	1	10	0.1	0.1	0.1	−1	−1	−1	118.35 ± 0.71	0.419 ± 0.011 ^d^

* Each experiment was performed in triplicate. Different small letters represent significant difference between different groups of numbers (*p* < 0.05).

**Table 3:** The regression results for the PB design.

Enzyme Activity					Biomass			
Factor	Coefficient Estimat	Effects	*p*-Value	*S*	Coefficient Estimat	Effects	*p*-Value	*S*
A	36.83	73.66	<0.0001	**	0.021	0.041	<0.0001	**
B	8.17	16.33	0.0138	**	0.0071	0.014	0.0084	**
C	12.50	24.99	0.0016	**	0.0066	0.013	0.0119	**
D	−1.38	−2.75			−0.0008	−0.00017		
E	20.29	40.57	<0.0001	**	−0.022	−0.044	<0.0001	**
F	3.45	6.90			0.0014	0.0028		
G	2.68	5.36			0.0011	0.0022		
H	3.79	7.58			−0.0026	−0.0052		
Dummy	−2.10	−4.20			0.0021	0.0042		
Dummy	−1.47	−2.95			−0.0024	−0.0048		
Dummy	1.35	2.69			−0.0026	−0.0052		

* Significant at *p* < 0.05; ** Significant at *p* < 0.01.

**Table 4:** Experiment design and result of response surface analysis.

Run	*X*_1_	*X*_2_	*X*_3_	*X*_4_	EA (U/mL)	BM (OD_600_)
1	−1	−1	0	0	240.99 ± 1.33	0.405 ± 0.006
2	1	−1	0	0	174.26 ± 1.80	0.448 ± 0.010
3	−1	1	0	0	217.43 ± 0.86	0.386 ± 0.008
4	1	1	0	0	241.56 ± 1.71	0.39 ± 0.007
5	0	0	−1	−1	150.97 ± 1.55	0.472 ± 0.011
6	0	0	1	−1	218.21 ± 1.42	0.481 ± 0.004
7	0	0	−1	1	217.54 ± 0.90	0.483 ± 0.023
8	0	0	1	1	105.86 ± 0.86	0.494 ± 0.006
9	−1	0	0	−1	180.70 ± 0.82	0.447 ± 0.005
10	1	0	0	−1	235.65 ± 1.38	0.488 ± 0.012
11	−1	0	0	1	250.14 ± 2.00	0.481 ± 0.008
12	1	0	0	1	140.93 ± 0.94	0.469 ± 0.008
13	0	−1	−1	0	235.93 ± 0.69	0.422 ± 0.006
14	0	1	−1	0	200.45 ± 1.07	0.387 ± 0.013
15	0	−1	1	0	188.06 ± 1.06	0.431 ± 0.020
16	0	1	1	0	212.07 ± 1.16	0.398 ± 0.009
17	−1	0	−1	0	194.38 ± 1.00	0.443 ± 0.009
18	1	0	−1	0	230.18 ± 1.21	0.451 ± 0.008
19	−1	0	1	0	220.04 ± 1.41	0.455 ± 0.015
20	1	0	1	0	146.33 ± 0.97	0.493 ± 0.021
21	0	−1	0	−1	185.89 ± 1.69	0.439 ± 0.011
22	0	1	0	−1	208.25 ± 0.49	0.380 ± 0.007
23	0	−1	0	1	163.21 ± 1.94	0.440 ± 0.008
24	0	1	0	1	193.42 ± 0.73	0.430 ± 0.005
25	0	0	0	0	270.32 ± 1.05	0.519 ± 0.018
26	0	0	0	0	271.85 ± 0.62	0.520 ± 0.020
27	0	0	0	0	269.09 ± 0.97	0.521 ± 0.015
28	0	0	0	0	260.33 ± 1.12	0.513 ± 0.011
29	0	0	0	0	259.36 ± 0.89	0.516 ± 0.017

* Each experiment was performed in duplicate.

**Table 5:** ANOVA for response surface polynomial model of all independent variables.

Factor	EA			Biomass		
	SS	*F*-Value	*Pr* > *F*	SS	*F*-Value	*Pr* > *F*
X_1_	1513.02	6.577711	0.0027 **	0.00124	29.75	<0.0001 ***
X_2_	597.81	0.795231	0.0383 *	0.00382	91.54	<0.0001 ***
X_3_	1607.21	6.359724	0.0022 **	0.00074	17.66	0.0009 **
X_4_	985.64	4.124658	0.0108 *	0.000675	16.19	0.0013 **
X_1_X_2_	2065.79	11.36978	0.0008 **	0.00038	9.12	0.0092 **
X_1_X_3_	2997.89	12.10719	0.0002 **	0.00023	5.40	0.0357 *
X_1_X_4_	6736.72	46.20569	<0.0001 ***	0.00070	16.85	0.0011 **
X_2_X_3_	885.00	6.160962	0.0147 *	0.000001	0.024	0.8791
X_2_X_4_	14.64	0.560155	0.7258	0.00060	14.40	0.0020 **
X_3_X_4_	8003.63	43.95054	<0.0001 ***	0.000001	0.024	0.6521
X_1_^2^	3148.00	11.63744	0.0001	0.00656	157.43	<0.0001
X_2_^2^	3562.08	23.78141	<0.0001	0.043	1041.34	<0.0001
X_3_^2^	10,960.79	63.65951	<0.0001	0.00407	97.70	<0.0001
X_4_^2^	16,077.00	94.12138	<0.0001	0.00115	29.75	0.0001
Model	49,077.94	19.7224	<0.0001 ***	0.054	92.78	<0.0001 ***
Residual error	1600.21			0.000583		
Lack of fit	1461.78	3.162004	0.0888	0.000521	3.32	0.0598
Pure error	138.43			0.000063		
Cor Total	50,678.15			0.055		

* Significant at *p* < 0.05; ** Significant at *p* < 0.01; *** Significant at *p* < 0.001.

**Table 6:** Logistic’ model parameters U_m_ (OD_600_/h), *X*_0_ (/OD_600_), *X_m_* (/OD_600_) and Luedeking-Piret’ model parameters α and β coefficients of determination (*R*^2^) of fitted models for all the treatments.

	Biomass					Enzyme Activity		
	Value	*U_m_*	*X*_0_	*X_m_*	*R*^2^	α	β	*R*^2^
**RS (r/min)**	80	0.186	0.017	0.396	0.976	352.453	6.770	0.979
	130	0.201	0.021	0.567	0.978	95.433	42.558	0.969
	200	0.214	0.017	0.451	0.988	106.824	46.014	0.971
**T (/°C)**	10	0.198	0.009	0.329	0.96	458.271	0.000	0.993
	20	0.257	0.008	0.553	0.972	300.319	20.764	0.961
	30	0.217	0.014	0.577	0.974	280.334	23.532	0.97
**IS (/mL)**	2	0.255	0.007	0.504	0.978	341.826	17.908	0.96
	3	0.214	0.016	0.655	0.976	330.606	16.432	0.981
	4	0.190	0.026	0.726	0.968	262.767	13.541	0.979

**Table 7:** Factors and levels of response surface analysis.

Level	Factor			
	X_1_: κ-Carrageenan g/L	X_2_: NaCl g/L	X_3_: Yeast Extract g/L	X_4_: FOS g/L
−1	1	15	0.5	0.5
0	2	20	1	1
1	3	25	1.5	1.5
